# Dosimetric characterization for GRID collimator‐based spatially fractionated radiation therapy: Dosimetric parameter acquisition and machine interchangeability investigation

**DOI:** 10.1002/acm2.14410

**Published:** 2024-05-29

**Authors:** Zhengzheng Xu, Salim Balik, Kaley Woods, Zhilei Shen, Chihyao Cheng, Jing Cui, Haihong Gallogly, Eric Chang, Lauren Lukas, Andrew Lim, Yutaka Natsuaki, Jason Ye, Lijun Ma, Hualin Zhang

**Affiliations:** ^1^ Keck School of Medicine University of Southern California Los Angeles California USA; ^2^ Department of Radiation Oncology University of Southern California Norris Comprehensive Cancer Center Los Angeles California USA

**Keywords:** GRID collimator, GRID therapy, peak valley dose ratio, spatially fractionated radiation therapy

## Abstract

**Purpose:**

The purpose of this study is to characterize the dosimetric properties of a commercial brass GRID collimator for high energy photon beams including 15 and 10 MV. Then, the difference in dosimetric parameters of GRID beams among different energies and linacs was evaluated.

**Method:**

A water tank scanning system was used to acquire the dosimetric parameters, including the percentage depth dose (PDD), beam profiles, peak to valley dose ratios (PVDRs), and output factors (OFs). The profiles at various depths were measured at 100 cm source to surface distance (SSD), and field sizes of 10 × 10 cm^2^ and 20 × 20 cm^2^ on three linacs. The PVDRs and OFs were measured and compared with the treatment planning system (TPS) calculations.

**Results:**

Compared with the open beam data, there were noticeable changes in PDDs of GRID fields across all the energies. The GRID fields demonstrated a maximal of 3 mm shift in dmax (Truebeam STX, 15MV, 10 × 10 cm^2^). The PVDR decreased as beam energy increases. The difference in PVDRs between Trilogy and Truebeam STx using 6MV and 15MV was 1.5% ± 4.0% and 2.1% ± 4.3%, respectively. However, two Truebeam linacs demonstrated less than 2% difference in PVDRs. The OF of the GRID field was dependent on the energy and field size. The measured PDDs, PVDRs, and OFs agreed with the TPS calculations within 3% difference. The TPS calculations agreed with the measurements when using 1 mm calculation resolution.

**Conclusion:**

The dosimetric characteristics of high‐energy GRID fields, especially PVDR, significantly differ from those of low‐energy GRID fields. Two Truebeam machines are interchangeable for GRID therapy, while a pronounced difference was observed between Truebeam and Trilogy. A series of empirical equations and reference look‐up tables for GRID therapy can be generated to facilitate clinical applications.

## 1 INTRODUCTION

Spatially fractionated radiotherapy (SFRT) with megavoltage x‐ray beams continues to gain popularity, as it has shown high rates of clinical response with minimal toxicities in treating large‐volume primary (e.g., diameter larger than 6 cm), metastatic, or refractory malignancies.^1^, ^2^, ^3^, ^4^, ^5^, ^6^, ^7^, ^8^, ^9^, ^10^, ^11^, ^12^ Currently, there are at least six known SFRT clinical trials going on, which are either Phase I of II for using GRID or Lattice Therapy in bulky tumor management.^13^ In addition, supported by the Radiosurgery Society (RSS), the GRID, Lattice, Microbeam and FLASH Radiation Therapy Working Groups (RSS GLMF Working Groups), Mayr et al.^8^ and Amendola et al.^14^ published international consensus guidelines to facilitate clinical trial design and multi‐institutional clinical trial collaboration for advanced head and neck, sarcoma, and gynecologic cancer with SFRT.

One way to deliver SFRT is to mount a physical GRID block (i.e., the GRID collimator) on the medical linear accelerator (Linac) accessory mount to generate the GRID collimator‐based SFRT beams. In addition to the physical blocks, the multi‐leaf collimators (MLCs) can also be used to generate cubical or cylindrical GRIDs and simulate two‐dimensional GRID therapy dosimetry.^15^ In a recent study, Zhang et al.^16^ reported that GRID collimator‐based SFRT delivers consistent heterogeneous doses and high‐dose core density across bulky tumor plans, which may be advantageous for multi‐institutional clinical trials. Currently, two types of GRID collimators are commercially available: (1) the High‐Dose Radiation GRID collimator (Radiation Products Design, Albertville, Minnesota, USA) that is constructed by casting divergent holes in a Cerrobend block^17^; (2) the Dotdecimal GRID collimator (.decimal Inc., Sanford, Florida, USA) that is made of a brass block with similar aperture patterns.^18^, ^19^ Because both types of GRID collimators use similar aperture designs, their dose profiles are similar and reproducible. Therefore, Zhang et al.^20^ developed the dosimetric parameter reference tables of the brass block collimator, including the peak/valley dose ratios (PVDRs) and output factors (OFs) across different depths and field sizes for 6 MV GRID fields. The lookup table enabled GRID therapy planning without the treatment planning system (TPS). The application of the lookup table assumes consistent GRID collimators design (e.g., collimator block thickness and holes arrangement pattern) and dosimetric parameters. With the intent of using higher energy beams for more complicated treatments, corresponding dosimetric parameters of the GRID fields must be verified before implementing the lookup table method.^20^ As the TPS can also be used for GRID therapy planning, a proper commissioning and experimental verification becomes crucial for ensuring TPS calculation accuracy. In order to calculate the dose of the GRID field in TPS, additional configurations are needed, including importing the DICOM file from the vendor that contains the geometric features of the GRID collimator, TPS configuration, and treatment console configuration.

With the adoption of high dose and the complex nature of SFRT, either using the look‐up table or TPS for treatment planning, it is critical to verify the accuracy and consistency of dosimetric properties of the GRID fields.^21^ Currently, most of the clinical applications or studies on SFRT using GRID collimator or Lattice therapy focused on the 6 MV photon beam. This is mainly due to the concern on neutron production from higher energy beams (i.e., > 6 MV). Mohiuddin et al. reported the dosimetric parameters of GRID beams with 6MV at depth of 5 cm only and the heterogeneity characteristics (e.g., PVDR) of the GRID beam were not reported.^22^ There are studies on high energy GRID field, however, the comprehensive dosimetric characteristics have not been reported. Meigooni et al. reported the output of the GRID beams on Varian Clinac21EX (6MV and 18MV) of various field sizes at dmax only and beam profiles of 10 × 10 cm^2^ at various depths.^17^ Beyer reported the GRID beam dosimetric parameters, including dmax, PDD10, TPR20/10, and beam profiles of 10 × 10 cm^2^ and 40 × 40 cm^2^ at depths of dmax and 10 cm for Varian's Trilogy and Clinac linacs. However, the output reported were measured only at 5 cm and the heterogeneity characteristics (e.g., PVDR) of the GRID beam were not reported.^23^ Zhang et al. reported more detailed dosimetric characteristics for the GRID beam of 6MV, including PDD, PVDR, and OFs for various field sizes and depths.^20^ However, there is lack of detailed comprehensive commissioning report of GRID collimator for newer model linacs and high energies.

Some studies reported that the neutron generation of high‐energy x‐ray beams is minimal from GRID collimators or MLC^24^, ^25^, ^26^, ^27^. In addition, a cross comparisons of the dosimetric parameters between machines (e.g., Trilogy vs. Trubeam, new vs. old versions of Truebeams) is clinically important for validating the interchangeability of different linacs. In this study, we aimed to fill these important knowledge gaps through dosimetrically characterizing the brass GRID collimator using low (6 MV) and high (10 and 15 MV) energy beams on Varian's Truebeam STX, Truebeam, and Trilogy machines (Varian Medical Systems, Inc., Palo Alto, California, USA). This is the first study that performed the comprehensive commissioning for GRID beams following the RSS GLMF working group guidelines. In addition, this study compared the measured dosimetric parameters of GRID beams using both low and high energies among three different linacs for potential machine exchangeability. This study further evaluated the GRID beam dose calculation accuracy using the TPS.

## 2 METHODS

### 2.1 Brass GRID collimator

**FIGURE 1:**
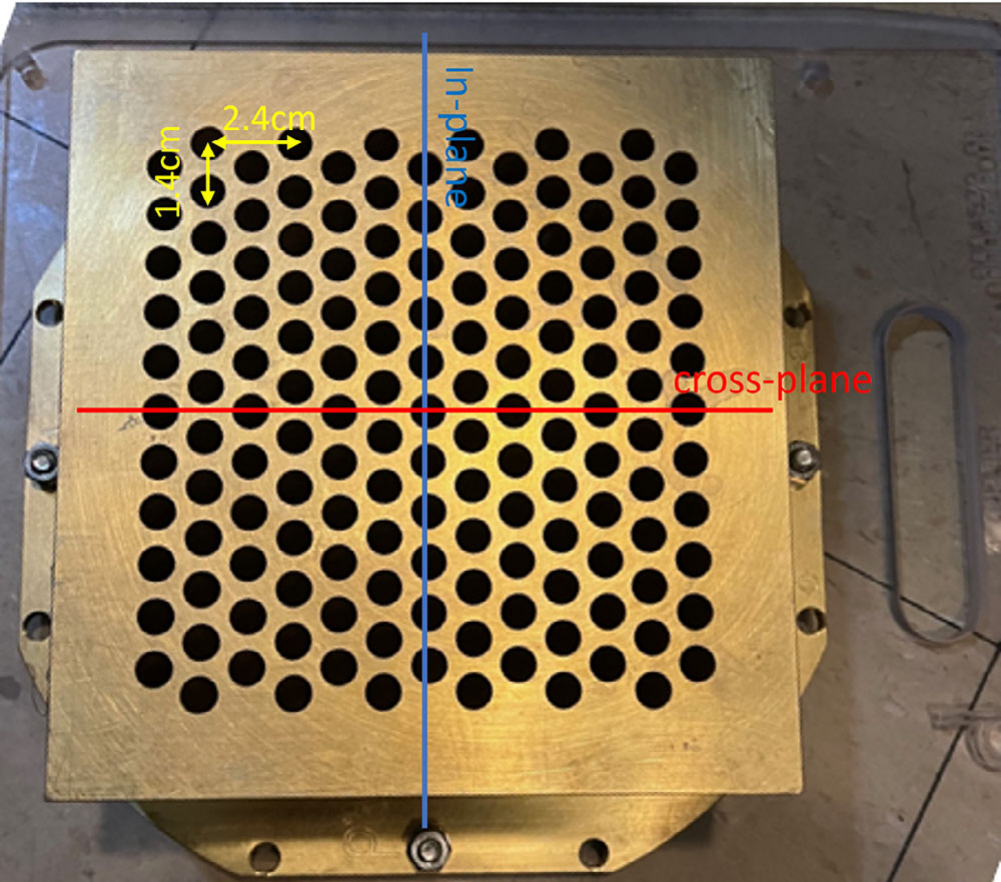
GRID collimator used in this study (DotDecimal, Sanford, Florida).

The GRID therapy has some unique dosimetric parameters as it deliberately creates a heterogeneous dose distribution.^18^ Therefore, the design of the GRID collimator is different from the traditional block or cutout used for photon or electron beams. The commercial GRID collimator is a brass block of 7.6 cm in thickness (Figure 1). The dosimetric characteristics of the GRID collimator block have been reported by several studies.^17^, ^19^, ^20^, ^21^, ^22^, ^23^ The holes are of 0.8 cm diameter on the upstream side, and they project about 1.00 cm diameter circular field at the isocenter. The center‐to‐center in‐plane and cross‐plane separations of the holes are 2.4  and 1.4 cm on the lower surface, respectively (Figure 1). The GRID collimator is attached to a plastic tray that can be inserted in the electron cone accessory mount and irradiate a maximum field size of 25 cm × 25 cm at 100 cm source to surface distance (SSD).

### 2.2 Water tank measurements

The characterization of the GRID field includes: (1) measuring the percent depth dose (PDD) profiles; (2) measuring the depths of maximum percent depth dose (dmax) for different field sizes; (3) acquiring the inline and crossline dose profiles at different depths; (4) measuring the peak to valley dose ratios (PVDR) at different depths with two field sizes (10 × 10 cm^2^ and 20 × 20 cm^2^); (5) measuring the OFs of 10 × 10 cm^2^ and 15 × 15 cm^2^ field sizes at different depths. All the above measurements were performed for 6, 10, and 15 MV.

This study followed the guidelines from RSS MLGF Working Groups for GRID beam commissioning.^18^ A PTW BEAMScan water phantom (PTW, Freiburg, Germany) and a PTW 60019 micro‐diamond detector (PTW, Freiburg, Germany) were used for the measurements of the GRID fields of 6, 10, and 15 MV of Varian's Truebeam and Truebeam STx linacs; and 6 and 15 MV of Varian's Trilogy linac. For simplicity, we labeled the Truebeam STX, Truebeam, and Trilogy linacs as STX, TB, and TRI, respectively. The measurements were performed at 100 cm SSD. The dmax were achieved from PDD of beams with 10 × 10 cm^2^ and 20 × 20 cm^2^. The beam profiles (in‐plane and cross‐plane) were acquired for beams with 10 × 10 cm^2^ and 20 × 20 cm^2^ at depths of dmax, 3 , 5 , 7 , 10 , and 15 cm. The OFs were measured for field sizes of 10 × 10 cm^2^ and 15 × 15 cm^2^, at depth of dmax, 3 , 5  7 , 10 , and 15 cm. The detector was aligned to the central hole of the GRID collimator for PDDs and OFs. All the measured data from three linacs were compared with each other and compared with the calculations using Eclipse TPS (v15.6, Varian Medical Systems, USA).

The peaks and valleys of the beam profile are dependent on the design of the collimator apertures. With the hexagonal honeycomb‐like hole pattern, the peak‐to‐peak distances are different along the inline and crossline directions, so are the valley doses. Following published studies, the PVDR is defined as

(1) $$\;{\mathrm{PVDR}}_{depth}=\bar{D_{depth}^{Peak}}\;/\bar{D_{depth}^{valley}}$$

where $\bar{D_{depth}^{Peak}}\;$ and $\bar{D_{depth}^{valley}}$ represent the averaged peak and valley doses (across the whole beam profile) at specific depth, respectively.^15^, ^16^

The OF of a GRID field is defined as the output of GRID field, $M_{GRID}\left(FS,d\right)$, of specific field size (FS) at depth (*d*), normalized to the output, $M_{open}(10x10,dmax$), a 10 × 10 cm^2^ open field at dmax at 100 cm SSD as recommended by the TG51 guideline.^28^

(2) $$\mathrm{OF}\left(FS,d\right)=\frac{M_{GRID}\left(FS,d\right)}{M_{open}\left(10x10,dmax\right)}\;$$

As the TB, STx, and TRI linacs are widely used in modern clinics, the measurements can be used to generate a reference dosimetric table for quick planning. Then, the monitor unit (MU) needed to deliver the prescription dose at depth can be calculated by

(3) $$\mathrm{MU}\left(FS,d\right)\;=\;\frac{\mathrm{Prescription}\;\mathrm{Dose}\;\left[cGy\right]}{OF\left(FS,\;d\right)*D_{o}\left(10x10,d_{max}\right)\;\left[\frac{cGy}{MU}\right]}$$

where $D_{o}\left(10x10,d_{max}\right)$ is the dose in cGy per MU of the user's beam under calibration condition. In this study, $D_{o}\left(10x10,d_{max}\right)$ of all the energies on three machines were calibrated to 1 cGy/MU at dmax at 100 cm SSD. We verified Equation 3 with the CC04 ion chamber (IBA dosimetry, USA) in a solid water phantom.

Varian's Truebeam linacs are now widely deployed in modern clinics and they commonly use a representative reference beam data set, also known as the “golden beam data.” As the beam data used in this study are in good agreement with the reference data from the vendor (i.e., within 1% or 1 mm), the measurements presented in this study may be suitable for generating a reference dosimetric table.

### 2.3 TPS dose calculations

Some modern TPS, such as Eclipse (v15.0 and higher), are capable of calculating the dose when using the GRID collimator. To commission the collimator model for TPS, the DICOM files provided by the vendor with geometric features were first imported into Eclipse (v15.6, Varian Medical Systems, Palo Alto, California, USA). A series of test plans, using the same beam geometric settings for water tank measurements, were generated with the imported GRID collimator in a 30 × 30 × 30 cm^3^ virtual solid water phantom. The GRID collimator properties in the TPS must agree with the actual GRID design (including the tray factor and block thickness) and its actual mounted position. The TPS calculation resolution was set to 1 mm. The TPS calculated dosimetric properties, including PDDs, dose profiles, PVDRs, and OFs, were compared with those obtained from water tank measurements.

### 2.4 Statistical analysis method

The Wilcoxon signed‐rank test^29^ was performed with Excel (Microsoft 2022, USA) for statistical analysis of the dosimetric differences among linacs and differences between measurements and TPS calculations. A *p*‐value of less than 0.05 was considered statistically significant. Polynomial regressions were performed to model the relationships between PVDR and depths for two field sizes and three linacs.

## 3 RESULTS

### 3.1 Water tank measurements

#### 3.1.1 PDDs

**FIGURE 2:**
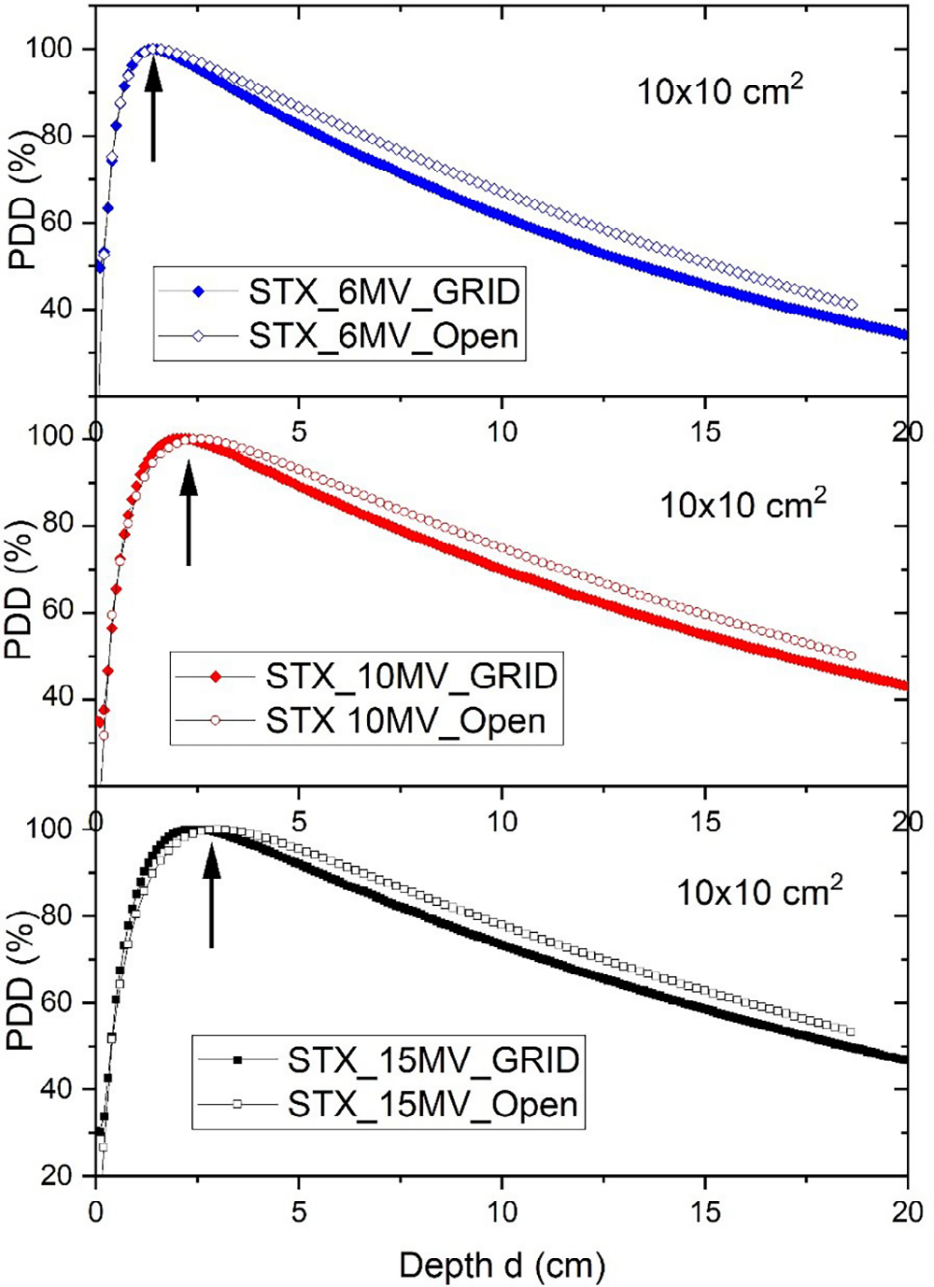
Measured PDDs for open and GRID fields on STX with 6, 10, and 15 MV beams. Data from STX was presented as an example. The changes in dmax were marked with arrows.

Figure 2 demonstrates the measured PDDs of STX, and the details of all the linacs are presented in Table 1. Compared to the dmax of open fields, those of the GRID beams were shallower due to the increased scattering radiation and energy degrading. For all the linacs, the changes in dmax for 6 and 10 MV beams were within 2 mm. The 15 MV 10 × 10 cm^2^ field on STX demonstrated the largest change in dmax of 3 mm (Table 2).

**TABLE 1:** Percentage depth dose data of 10 × 10 cm^2^ open and GRID fields on three linac machines.

	TRI		STX		TB	
	Open	GRID	Open	GRID	Open	GRID
	**6 MV**					
**Depth (cm)**	**PDD (%)**					
0.5	82.3	89.8	82.5	82.3	84.1	85.8
1	98.0	99.4	98.0	98.0	97.9	98.7
1.5	99.9	99.5	99.9	100.0	100.0	99.9
2	98.8	97.2	98.9	98.0	98.8	98.0
3	94.9	91.9	95.0	92.8	94.8	92.8
4	90.7	86.8	90.8	87.7	90.6	87.6
5	86.4	81.9	86.6	82.6	86.5	82.5
7	78.5	73.0	78.5	73.6	78.2	73.5
10	67.2	61.3	67.0	61.6	66.7	61.4
12	60.1	54.6	60.0	54.6	59.7	54.6
14	53.9	48.6	53.7	48.5	53.4	48.6
15	51.1	45.6	50.8	45.6	50.6	45.7
	**10 MV** ^a^					
**Depth (cm)**	**PDD (%)**					
0.5	–	–	69.2	65.4	72.9	70.0
1	–	–	88.2	89.1	90.2	91
1.5	–	–	96.1	97.8	97.1	98.3
2	–	–	99.1	99.9	99.5	100.0
3	–	–	99.5	97.8	99.1	97.6
4	–	–	96.6	93.5	96.0	93.4
5	–	–	93.0	89.1	92.2	88.9
7	–	–	85.5	80.9	85.2	80.7
10	–	–	74.9	69.9	74.5	69.9
12	–	–	68.4	63.6	68.1	63.5
14	–	–	62.4	57.7	61.7	57.9
15	–	–	59.6	54.8	59.0	54.7
	**15 MV**					
**Depth (cm)**	**PDD (%)**					
0.5	64.1	71.2	63.9	60.8	66.0	65.3
1	84.8	90.0	84.1	85.0	84.7	87.2
1.5	93.7	97.1	93.1	95.6	93.5	96.5
2	97.9	99.6	96.8	99.4	97.8	99.5
3	100.0	98.8	99.9	99.3	100.0	99.2
4	98.2	95.7	98.5	96.1	98.1	95.8
5	95.2	91.9	95.4	92.0	95.0	91.9
7	88.0	83.7	88.3	84.0	87.8	83.8
10	77.7	73.2	77.9	73.4	77.4	73.3
12	71.3	67.0	71.5	67.1	71.0	67.0
14	65.3	61.1	65.5	61.3	65.1	61.1
15	62.5	58.5	62.8	58.5	62.3	58.4

^a^ The 10MV beam is not available on Varian's Trilogy linac.

**TABLE 2:** Depths of the maximum percentage dose (dmax) of the open and GRID fields with different field sizes on different machines.

	10 × 10 cm^2^ TPS	10 × 10 cm^2^ Measured	10 × 10 cm^2^ Measured	20 × 20 cm^2^ TPS	20 × 20 cm^2^ Measured	20 × 20 cm^2^ Measured
Energy (MV)	Open (cm)	Open (cm)	GRID (cm)	Open (cm)	Open (cm)	GRID (cm)
	**TRI** ^a^					
6	1.3	1.4	1.2	1.5	1.4	1.3
10	–	–	–	–	–	–
15	2.4	2.4	2.4	2.5	2.4	2.4
	**STX**					
6	1.4	1.5	1.4	1.5	1.5	1.5
10	2.3	2.3	2.2	2.4	2.3	2.3
15	2.6	2.8	2.5	2.8	2.7	2.7
	**TB**					
6	1.4	1.5	1.4	1.5	1.5	1.5
10	2.2	2.3	2.2	2.3	2.3	2.3
15	2.5	2.7	2.5	2.6	2.6	2.6

^a^ The 10MV beam is not available on Varian's Trilogy linac.

#### 3.1.2 GRID Beam profiles and PVDR

**FIGURE 3:**
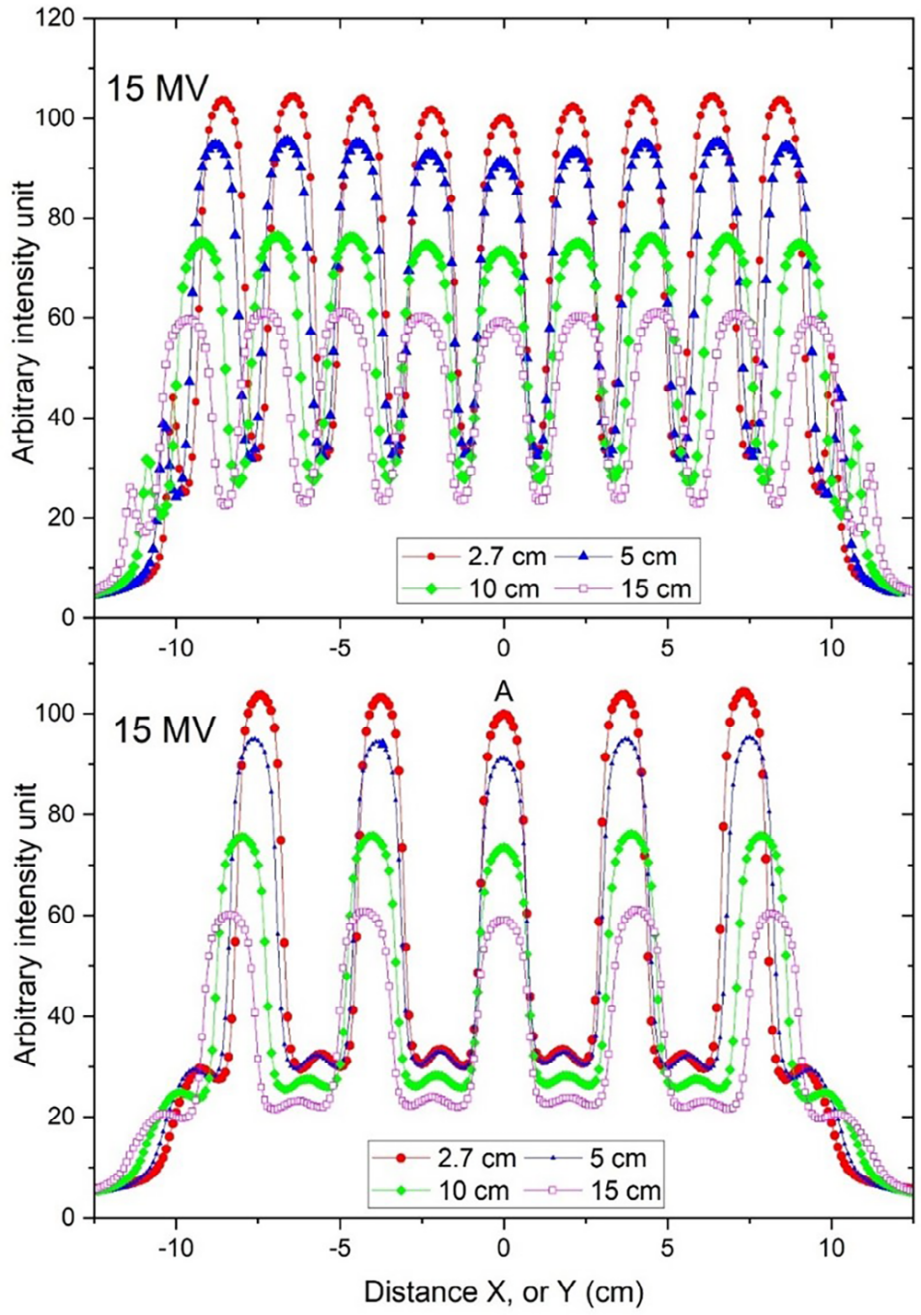
Dose profiles of a 20 × 20 cm^2^ GRID field using 15 MV on STX at various depths. All profiles were normalized to the central peak value at dmax.

The profiles of 6 MV GRID field agreed with the published data.^20^ For illustration, dose profiles of the 15 MV GRID field with 20 × 20 cm^2^ field size on STX are shown in Figure 3. As the beam is flattened at 10 cm depth, the peak values of the GRID field were more consistent at 10 cm compared to those at shallower depths. The profiles of other energies were of the similar pattern.

**FIGURE 4:**
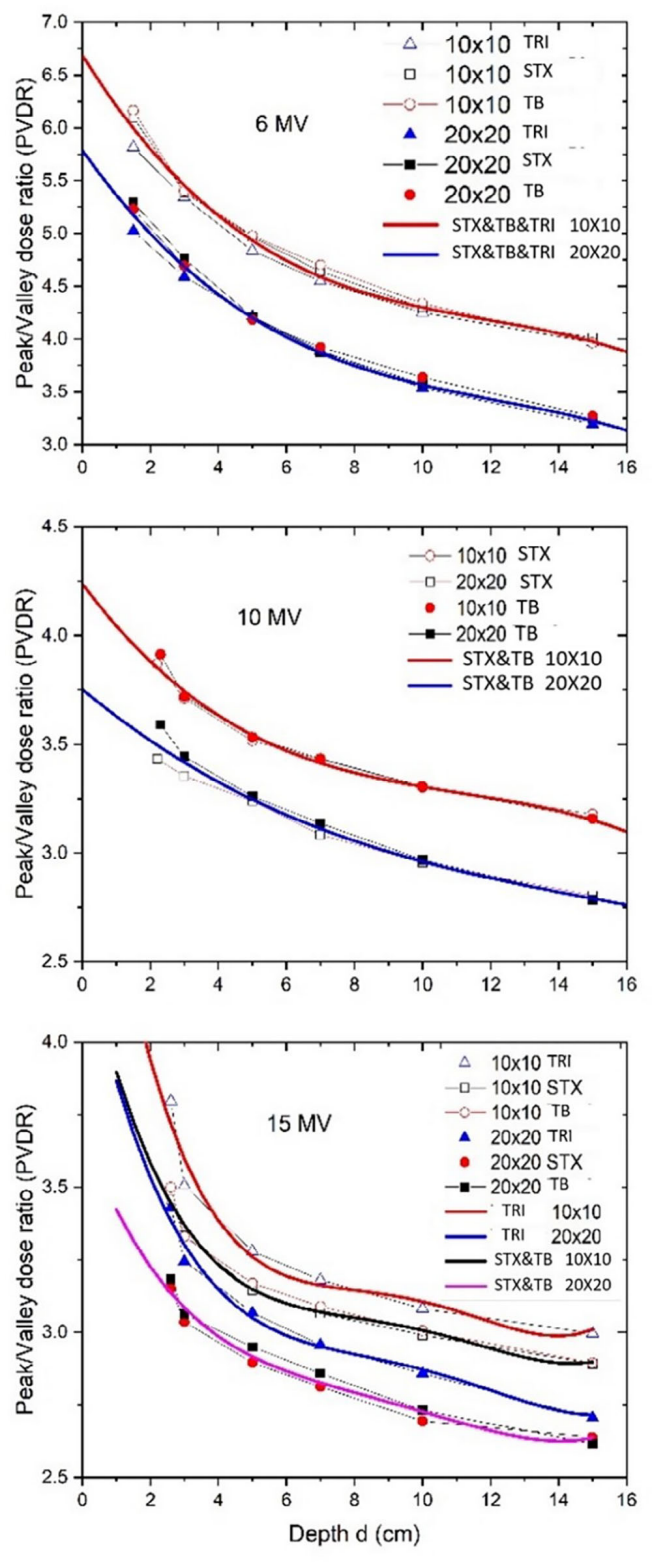
PVDRs versus depth, and corresponding polynomial fittings for GRID fields of different geometries on different linacs.

Table 3 and Figure 4 demonstrate the correlation between PVDRs, depth, field sizes, and energies. It was found that the difference in measured PVDRs between the STX and TB linacs at a given depth was negligible (*p* = 0.12). However, there was noticeable difference in PVDRs between the TRI and Truebeams (i.e., STX/TB) linacs (*p* = 0.03).

**TABLE 3:** Averaged PVDRs for TRI, STX, and TB^a^.

	TRI		STX		TB	
Average	10 × 10	20 × 20	10 × 10	20 × 20	10 × 10	20 × 20
	6 MV					
**Depth (cm)**						
1.5	5.82	5.03	6.10	5.30	6.17	5.23
3	5.34	4.59	5.39	4.76	5.40	4.69
5	4.84	4.21	4.97	4.21	4.98	4.18
7	4.55	3.89	4.64	3.88	4.70	3.92
10	4.25	3.54	4.30	3.57	4.34	3.64
15	3.97	3.20	4.01	3.24	3.97	3.27
	10 MV^b^					
**Depth (cm)**						
2.2	–	‐	3.87	3.43	3.91	3.59
3	–	‐	3.71	3.35	3.72	3.44
5	–	‐	3.52	3.24	3.53	3.26
7	–	‐	3.43	3.08	3.43	3.14
10	–	‐	3.30	2.96	3.31	2.97
15	–	‐	3.18	2.80	3.16	2.79
	15 MV					
**Depth (cm)**						
2.6	3.80	3.43	3.46	3.15	3.50	3.18
3	3.51	3.25	3.33	3.04	3.33	3.06
5	3.28	3.07	3.15	2.90	3.17	2.95
7	3.18	2.96	3.06	2.81	3.09	2.86
10	3.08	2.86	2.99	2.69	3.01	2.73
15	3.00	2.71	2.89	2.64	2.89	2.62

^a^ PVDR was the average of both in‐plane and cross‐plane profiles.

^b^ The 10MV beam is not available on Varian's Trilogy linac.

The polynomial function fitting was generated and validated for PVDRs as a function of depth (*d*) in cm for GRID fields of 10 × 10 cm^2^ or 20 × 20 cm^2^ (Equation 4) using software R (R Core Team, 2023). PVDR of a particular field size at any given depth can be calculated by the following equation (Equation 4). The fitting coefficients are listed in Table 4.

(4) $$\begin{array}{ccc} \mathrm{PVDR}\;\left(d\right) & = & \;a4\times d^{4}+a3\times d^{3}\\ & & +\;a2\times d^{2}+a1\times d+a0 \end{array}$$

**TABLE 4:** PVDR foruth and third‐order polynomial fitting function coefficients for different machines and beam energies at various GRID field sizes (Equation 4).

Energy	Field size (cm x cm)	a4	a3	a2	a1	a0
**TRI** ^a^						
6 MV	10 × 10		−1.07 × 10^−3^	3.85 × 10^−2^	−5.16 × 10^−1^	6.69
	20 × 20		−8.69 × 10^−4^	3.20 × 10^−2^	−4.56 × 10^−1^	5.79
15 MV	10 × 10	2.37 × 10^−4^	−9.37 × 10^−3^	1.34 × 10^−1^	−8.48 × 10^−1^	5.17
	20 × 20	1.33 × 10^−4^	−5.43 × 10^−3^	8.02 × 10^−2^	−5.38 × 10^−1^	4.33
**STX and TB**						
6 MV	10 × 10		−1.07 × 10^−3^	3.85 × 10^−2^	−5.16 × 10^−1^	6.69
	20 × 20		−8.69 × 10^−4^	3.20 × 10^−2^	−4.56 × 10^−1^	5.79
10 MV	10 × 10		−4.98 × 10^−4^	1.66 × 10^−2^	−2.10 × 10^−1^	4.24
	20 × 20		−1.39 × 10^−4^	6.48 × 10^−3^	−1.30 × 10^−1^	3.75
15 MV	10 × 10	1.43 × 10^−4^	−5.66 × 10^−3^	8.09 × 10^−2^	−5.20 × 10^−1^	4.34
	20 × 20	8.82 × 10^−5^	−3.30 × 10^−3^	4.58 × 10^−2^	−3.13 × 10^−1^	3.69

The 10MV beam is not available on Varian's Trilogy linac.

The PVDRs for 6 and 10 MV GRID fields could be approximated by the third‐order polynomial functions, while those for 15 MV beams could be better fitted with the fourth order polynomial functions (i.e., higher *R* ^2^ for the fitting model). For 6 MV beams, although there were differences in PVDRs at shallow depths among three linacs, the fitting models could still be considered as machine independent for two Truebeam linacs. For 10 and 15 MV GRID fields, both STX and TB demonstrated similar correlation between PVDR and depth. As shown in Table 4, there were two fitting models derived for 6 and 10 MV GRID fields (i.e., 10 × 10 cm^2^ and 20 × 20 cm^2^), and four models for 15 MV GRID fields (i.e., Truebeams and Trilogy; 10 × 10 cm^2^ and 20 × 20 cm^2^).

#### 3.1.3 Output factors

For all the investigated energies and field sizes, the OFs ranged from 0.937 to 0.420 with depths ranging from 1.5 to 15 cm (Table 5). There was no significant difference in OFs among three linacs. The difference between calculations and measurements was within 3% at any investigated depth.

**TABLE 5:** Measured OF for GRID beams on different machines^a^.

	TRI^b^		STX		TB	
	10 × 10	15 × 15	10 × 10	15 × 15	10 × 10	15 × 15
**Depth (cm)**	**6 MV**					
1.5	0.914	0.939	0.920	0.937	0.911	0.936
3.0	0.848	0.870	0.848	0.868	0.850	0.869
5.0	0.756	0.779	0.755	0.776	0.756	0.777
7.0	0.674	0.695	0.672	0.693	0.676	0.694
10.0	0.565	0.588	0.563	0.586	0.566	0.584
15.0	0.420	0.442	0.419	0.439	0.420	0.439
**Depth (cm)**	**10 MV**					
2.2	–	‐	0.869	0.895	0.874	0.896
3.0	‐	‐	0.849	0.871	0.854	0.876
5.0	‐	‐	0.773	0.796	0.780	0.801
7.0	‐	‐	0.703	0.726	0.708	0.729
10.0	‐	‐	0.608	0.630	0.612	0.633
15.0	‐	‐	0.476	0.498	0.480	0.499
**Depth (cm)**	**15 MV**					
2.6	0.870	0.896	0.853	0.878	0.858	0.885
3.0	0.865	0.887	0.844	0.870	0.851	0.876
5.0	0.800	0.820	0.781	0.804	0.789	0.810
7.0	0.732	0.751	0.715	0.735	0.722	0.742
10.0	0.638	0.657	0.624	0.645	0.630	0.649
15.0	0.506	0.523	0.500	0.517	0.501	0.520

^a^ OF was normalized by the 10 × 10 cm^2^ field at dmax.

^b^ The 10MV beam is not available on Varian's Trilogy linac.

### 3.2 Verifications of TPS dose calculation accuracy

**FIGURE 5:**
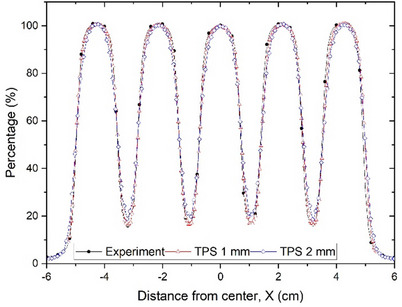
GRID beam profiles of TPS calculations (1 mm and 2 mm resolutions) and water tank measurement.

The measured PDD of GRID fields agreed well with the TPS calculations. The dmax from calculated PDD profiles were consistent with those acquired from measurements (Table 2). Compared with the TPS calculations using 1 mm resolution, dose profiles at different depths agreed with the measurements within ± 3% deviation. Figure 5 illustrates the measured GRID field profile and corresponding TPS calculated profiles using 1 and 2 mm resolutions, respectively.

Compared with the TPS calculation results, PVDRs and OFs of three linacs demonstrated less than 3% difference (*p* > 0.05). The difference in PVDRs between measurements and TPS calculations decreases with increasing depth. For example, the difference in PVDR between measured and TPS calculations of a 6MV GRID field on STX (10 × 10 cm^2^ at dmax) was 2.9% (measured:6.29 vs. calculated: 6.11), while it decreased to 0.2% at 7 cm depth (measured: 4.80 vs. calculated: 4.79). The measured OFs agreed with TPS calculations within 3%.

## 4 DISCUSSION

Before the adoption of MLC‐based SFRT, the commercial GRID collimators provided the first standardized and easily achievable SFRT for patients, which was a critical improvement for the initial in‐house produced GRID blocks.^30^, ^31^ When conducting a clinical trial for SFRT, the dosimetric consistency among participating institutions is important due to the heterogeneous nature of the GRID fields. There was a noticeable difference in valley doses between TRI and STX/TB, which resulted in different PVDRs. Beyer et al. reported the difference of 0.4 mm (at dmax) to 0.5 mm (at 10 cm) in beam penumbra for a beam of 15MV and 10 × 10 cm^2^ field size between Trilogy and Truebeam.^23^ Their finding on the difference in beam energy spectra between Trilogy and Truebeam supports the difference in GRID therapy dosimetry presented in this study. Difference in linac gantry head designs, materials, and spot size will affect the beam's scattering and the energy spectrum.^23^ Although some dosimetric parameters of the GRID field demonstrated certain machine dependence, the PDDs, cross‐plane profiles, and OFs were still consistent with the published results.^19^, ^20^, ^23^

All the measurements agreed with the TPS calculations within 3%, which was used as the action limit in the study by Zhang et al.^20^ When the TPS is not available, the OF is an essential parameter for GRID therapy hand calculation using the lookup table. In this study, the difference in OFs between measurements and TPS calculations was within 3%, which complies with the AAPM medical physics practice guidelines 5.a.^32^ It is important to ensure that the commissioning of GRID collimator follows the general linac commissioning protocol, such as the machine setup and normalization methods.

In addition to the dosimetric parameters presented in this study, there are still more details that need to be considered in clinical applications of the GRID collimator. As illustrated in Figure 3, due to the difference in holes’ arrangement, there was difference in valley doses between in‐plane and cross‐plane profiles.^20^, ^27^ The maximal difference (i.e., Valley_in‐plane/Valley_cross‐plane‐1)*100%) was found to be 7.9% for TRI 6 MV with 10 × 10 cm^2^ field size at dmax. PVDR will be affected when different profiles are used. Such discrepancy in PVDR due to the difference in valley doses between in‐plane and cross‐plane profiles was observed for all the beams. However, the difference in PVDR for beams with high energy (i.e., 10 or 15 MV) and large field sizes (e.g., 20 × 20 cm^2^) ranged just from 1.1% to 3.4%. The GRID collimator is normally used at a fixed gantry angle with collimator and MLC adjusted for target dose conformality. Since the aperture orientation is not included in the planning consideration, an averaged PVDR over both in‐plane and cross‐plane profiles is more appropriate in clinical application. Therefore, this study presented the averaged PVDRs over the in‐plane and cross‐plane profiles.

As the beamlet diameter projected at the isocenter plane is around 1 cm (full width of half maximum), an appropriate measurement protocol and a robust scanning system are critical for acquiring accurate dosimetric parameters for those small fields. The GRID field profiles will be significantly affected by the equipment selection and setup accuracy. Nowadays, the most advanced water tank systems can perform the leveling and detector positioning automatically to achieve the optimal alignment for minimizing the setup uncertainty. The micro‐diamond detector is proven to be an appropriate detector for measuring this heterogeneous, small beamlet‐packed field because of its superior features, including small effective volume, high‐dose response, and directional independence.^33^, ^34^ In addition, a slow to moderate scanning speed is recommended for acceptable measurement accuracy. The profile measurement accuracy could be compromised by water phantom perturbation if the detector moves too fast. Lastly, from our experience, the TPS calculation resolution of less than 2 mm is highly recommended for accurate GRID therapy dose calculation. A maximum of 8% difference in PVDR was found when using 2 mm or lower calculation resolution due to the volume averaging effect on the heterogeneous doses, especially in the valley regions.

When using GRID therapy to treat a large tumor, the depth dependency of PVDR may result in different dose levels across the target. Zhang et al. stated that single point peak and valley doses may not accurately represent the degree of spatial fractionation, thus the RSS GLMF Working Groups recommend using the dosimetry parameter of D10/D90, which is the ratio between the dose covering 10% and 90% of target volume.^18^

PVDRs of higher energies are less than 4.0, indicating a smaller dose modulation compared to GRID beams using 6 MV. Theoretically, higher PVDR is hypothesized to have less toxicity when using the same prescription dose for SFRT. Clements et al. used Monte Carlo simulation to calculate the dose for a customized GRID collimator and reported a PVDR of 5.0 for a 10 MV GRID field.^35^ The difference in PVDRs between Clements's study and our measurements is the differences is mainly due to the GRID aperture design, material compositions, and beam's energy spectrum. GRID beams of 15 MV have even lower PVDRs, indicating higher internal scattering radiation compared to those of 6 MV and 10 MV GRID fields. The empirical equations provided a method to calculate the PVDR for GRID fields of 6 MV, 10 MV and 15 MV with field sizes of 10 × 10 cm^2^ and 20 × 20 cm^2^, at depth ranging from 0.5 to 15 cm.

One limitation of this study is that the measurements were conducted in the standard setup where the beam's central axis was perpendicular to the water surface. In clinical treatment, there will be gantry rotation, irregular patient surface, and heterogeneity that may affect the dosimetric characteristics of the GRID fields. Further studies are needed to investigate the dosimetric impact due to the clinical settings.

## 5 CONCLUSION

With the same clinical setting, there is a consistent decrease in PVDR with increasing photon beam energy. A set of empirical equations and reference look‐up tables for PVDRs can be formulated during the commissioning to facilitate the treatment planning. Using high calculation resolution, the dosimetric parameters from TPS agreed well with the measurements. We validated the interchangeability of two Truebeam linacs, while the Trilogy linac still demonstrated noticeable differences. Therefore, a comprehensive commissioning procedure is critical for developing a safe and reliable GRID collimator based SFRT program.

## AUTHOR CONTRIBUTIONS

All authors contributed to the study conception, design, and manuscript drafts revisions.

## CONFLICT OF INTEREST STATEMENT

The authors declare no conflicts of interest.
